# A Recombinase-Based Genetic Circuit for Heavy Metal Monitoring

**DOI:** 10.3390/bios12020122

**Published:** 2022-02-16

**Authors:** Doğuş Akboğa, Behide Saltepe, Eray Ulaş Bozkurt, Urartu Özgür Şafak Şeker

**Affiliations:** UNAM-Institute of Materials Science and Nanotechnology, Bilkent University, Ankara 06800, Turkey; dogus.akboga@bilkent.edu.tr (D.A.); behide.saltepe@bilkent.edu.tr (B.S.); ulas.bozkurt@bilkent.edu.tr (E.U.B.)

**Keywords:** heavy metal detection, whole-cell biosensor, Bxb1 recombinase, synthetic biology, toxicity

## Abstract

Rapid progress in the genetic circuit design enabled whole-cell biosensors (WCBs) to become prominent in detecting an extensive range of analytes with promise in many fields, from medical diagnostics to environmental toxicity assessment. However, several drawbacks, such as high background signal or low precision, limit WCBs to transfer from proof-of-concept studies to real-world applications, particularly for heavy metal toxicity monitoring. For an alternative WCB module design, we utilized Bxb1 recombinase that provides tight control as a switch to increase dose-response behavior concerning leakiness. The modularity of Bxb1 recombinase recognition elements allowed us to combine an engineered semi-specific heat shock response (HSR) promoter, sensitive to stress conditions including toxic ions such as cadmium, with cadmium resistance regulatory elements; a cadmium-responsive transcription factor and its cognitive promoter. We optimized the conditions for the recombinase-based cadmium biosensor to obtain increased fold change and shorter response time. This system can be expanded for various heavy metals to make an all-in-one type of WCB, even using semi-specific parts of a sensing system.

## 1. Introduction

Environmental monitoring of toxicity generated by heavy metals has become a requisite concern due to rapidly growing industries such as metallurgy and agriculture. Heavy metals form solid covalent bonds with organic molecules, affecting cellular organelles and elements by causing irreversible DNA damage and inhibiting the cell cycle that leads to apoptosis and carcinogenesis [1]. Current assessment methods of heavy metal toxicity, including but not limited to sequential sedimentation [2], inductively coupled plasma mass spectrometry (ICP-MS) [3], atomic absorption spectrometry [4], and the use of indicator species [5], are analytical methods to detect single contamination at any given time and require expensive instruments as well as skilled personnel. On the other hand, the challenge in the proper toxicity assessment is the detection of the bioavailable fractions of heavy metals as monitoring bioavailability can further help measure the quantitative effects of these metals on the environment and be useful for their bioremediation [6].

Cell-based biosensors tackle this challenge by detecting bioavailable fractions of chemicals through intrinsic regulatory mechanisms. One of the first biosensors for heavy metal toxicology is luminescent marine bioassays that depend on aquatic strains of bacteria such as *Vibrio fischeri*. These bioassays effectively detect contaminants in environmental samples [7]; nevertheless, they are nonspecific, and their sensitivity is highly affected by physical factors and environmental variations [8,9]. In contrast, synthetic biology has advanced the concept of biosensing with the engineered whole-cell biosensors (WCBs) [10]. The WCBs approach is based on the concept of modules with biological parts that have been wired to obtain a digital logic gate response [11] and consists of sensing, processing, and output units [12,13]. Other than estimating the bioavailable fraction of heavy metals, WCBs can process complex heavy metal signals into easily detectable outputs; and modularity of the sensing parts provides opportunities to multiplex the detection of a spectrum of toxic metals [14] as well as to manipulate crosstalk [15], and sensitivity [16]. This modularity of biological parts facilitates the construction of semi-specific biosensors that utilize the stress response of microbial strains [17,18] and specific biosensors, in which a specific target molecule activates or represses the transcription of a particular output [19,20].

**Figure 1:**
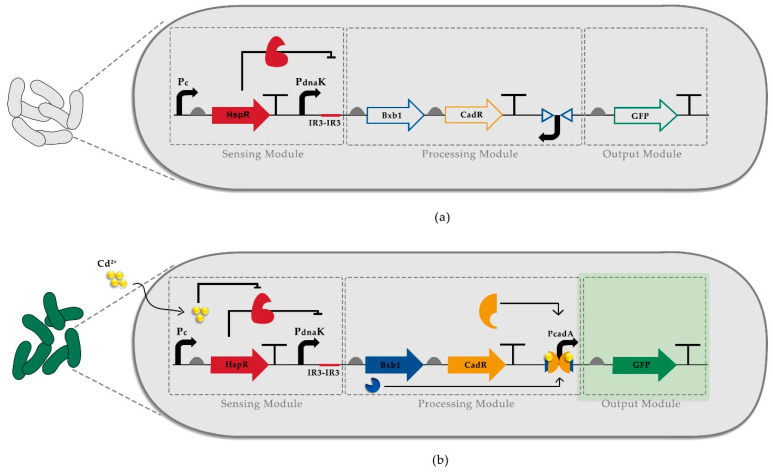
Recombinase-based whole-cell biosensor design. (**a**) HspR constantly represses the engineered stress promoter, PdnaK-IR3-IR3, in the absence of cadmium so that neither Bxb1 nor CadR is expressed, consequently controlling GFP expression. (**b**) The presence of cadmium ion causes stress-activating heat shock response, and HspR is released from the engineered stress promoter so that Bxb1 and CadR expression is initiated. In the first step, Bxb1 recombines the inverted PcadA promoter. Secondly, the CadR-cadmium complex initiates the reporter expression by binding to recombined PcadA. Pc; constitutively active promoter.

Furthermore, one of the essential tools of synthetic biology is recombinases, which are frequently used in logic gates [21]. By inverting/excising the target sequence flanked by recognition sites, recombinases provide an ON/OFF mechanism [22]. Hence, they could be used to oversee various biological parts to obtain a digital response. Recombinases are also incorporated into Boolean logic gates to enhance the specificity [23], and ON/OFF response can lower the background signal of a circuit-based biosensor when the activation of the promoter depends on the recombinase activity (Figure 1). Additionally, recombinases are used to build biosensors that can record environmental signals [24].

This study proposes a recombinase-based heavy metal biosensing system [25] that combines a semi-specific biosensing device, our previously engineered heat shock response (HSR) mechanism [26], with a specific biosensing module consisting of metal ion resistance genes. We used cadmium ion (Cd (II)) as our source to detect and used transcription factor, CadR, and leaky and cross-reactive efflux pump promoter, PcadA, from microorganism *Pseudomonas putida* [27]. By characterizing optimal conditions for this system based on Bxb1 recombinase [28], we also provide a guideline to comprehending the possible drawbacks of a recombinase-based whole-cell biosensor.

## 2. Materials and Methods

### 2.1. Strain and Growth Media

*E. coli* DH5α (New England Biolabs, Ipswich, MA, USA) strain, both used in plasmid construction and source detection assays, was grown in Lysogeny Broth (LB) medium (1% (*w*/*v*) tryptone, 0.5% (*w*/*v*) yeast extract, 0.5% (*w*/*v*) NaCl) with proper antibiotics. Overnight cultures were prepared from −80°C glycerol (25% (*v*/*v*)) stocks in 3 mL LB and cultivated in 37 °C incubator with shaking (200 rpm) (INNOVA 44, New Brunswick Scientific, Edison, NJ, USA).

To start experimental cultures, 0.4%, 1% and 2% of overnight inoculums were diluted in fresh LB, or MOPS minimal media (0.1 M potassium morpholinopropane sulfonate (MOPS), PH 7.4; 0.1 M Tricine, PH 7.4; 0.001 M FeSO_4_; 0.19 M NH_4_ Cl; 0.0276 M K_2 SO_4_; 0.002 CaCl_2_; 0.25 M MgCl_2_; 0.5 M NaCl; micronutrients [3 × 10^−2^ M (NH_4_)_6_ Mo_7_ O_24_; 4 × 10^−5^ M H_3_ BO_3_; 3 × 10^−6^ M CoCl_2_; 10^−6^ M CuSO_4_; 8 × 10^−6^ M MnCl_2_; 10^−6^ M ZnSO_4_]; 0.132 M K_2 HPO_4_; 1 mg/mL thiamine; supplemented with 0.2% (*v*/*v*) glucose) defined by Reference [29], or Heavy Metal MOPS (HMM) media (40 mM MOPS, PH 7.2; 50 mM KCl; 10 mM NH_4_ Cl; 0.5 mM MgSO_4_; 1 mM glycerol-2-phosphate (BGP); 1 μM FeCl_3_; supplemented with 0.4% (*v*/*v*) glucose) defined by Reference [30]. Each culture was inoculated in either 96-well microplates (353916, Corning) to a final volume of 250 µL/well or 15 mL falcons to a final volume of 3 mL/falcon and induced with proper inducers. Experimental cultures started with 2% inoculums were induced after their OD_600_ reached between 0.4–0.6. Cells were incubated at either 23 °C or 30 °C or 37 °C for a ranging time interval in stable incubators (INCU-Line, VWR) unless otherwise stated.

Antibiotics (34 µg/mL chloramphenicol, 100 µg/mL ampicillin, 100 µg/mL spectinomycin) and chemicals used in source detection assays (Na_3_ AsO_4_, CoCl_2_ 6H_2_O, PbCl_2_, Cd(OOCCH_3_)_2_ 2H_2_O) were analytical grade and purchased from Sigma-Aldrich (St. Louis, MO, USA). Each reagent was dissolved in ddH_2_O and filter sterilized using 0.22 µm syringe filters (16,532 K, Sartorius AG, Göttingen, Germany).

### 2.2. Sensor Plasmid Assembly

Standard molecular biology methods were used for the construction of the recombinase-based cadmium sensor plasmids. To construct the plasmid containing sensing and output modules (mProD HspR PcadA (inverted) GFP pet22 b vector), PcadA promoter with Bxb1 recognition sites were amplified using PCR with Q5 High Fidelity DNA polymerase (M0491, New England Biolabs) in a thermal cycler (C1000 Touch, Bio-Rad). Backbone was obtained from a previously constructed plasmid (mProD HspR PgolB (inverted) GFP pet22 b vector) [25] using PCR to exclude PgolB promoter. All pieces were run on 1% or 2% (*w*/*v*) Agarose gel (A9539-500G, Sigma-Aldrich, St. Louis, MO, USA) stained with SYBR Safe DNA stain (S33102, Invitrogen, Waltham, MA, USA). Bands at expected sizes were isolated using NucleoSpin Gel and PCR Clean-up kit (Macherey-Nagel). The plasmid was constructed via Gibson Assembly described by Reference [31]. After assembly, the entire mix was transformed into chemically competent *E. coli* DH5α cells.

To construct the plasmid containing processing module (PdnaK-IR3-IR3 Bxb1 CadR pZa vector), the previously constructed PdnaK-IR3-IR3 GFP pZa vector [26] was digested with MluI restriction endonuclease. The digestion product was run on 1% Agarose gel, and the band at the expected size was isolated using NucleoSpin Gel and PCR Clean-up kit (Macherey-Nagel). Bxb1 recombinase and CadR were amplified using PCR with Q5 High Fidelity DNA polymerase (M0491, New England Biolabs) in a thermal cycler (C1000 Touch, Bio-Rad). All pieces were run on 1% or 2% (*w*/*v*) Agarose gel stained with SYBR Safe DNA stain (S33102, Invitrogen). Bands at expected sizes were isolated using NucleoSpin Gel and PCR Clean-up kit (Macherey-Nagel). The plasmid was constructed via Gibson Assembly described by Reference [31]. After assembly, the entire mix was transformed into chemically competent *E. coli* DH5α cells.

Primers used in this study were purchased from PRZ Biotech and listed in Table S1. All genetic parts used in this study are summarized in Table S2. Plasmid map representations are provided in Figure S1. Constructed plasmids were verified by Sanger Sequencing (GENEWIZ).

### 2.3. Fluorescence Measurement and Data Analysis

For dynamic range analysis, experimental cultures were induced with 0–100 or 0–250 µM of cadmium ions, arsenic ions, or lead ions. All fluorescence measurement studies were conducted via microplate reader (SpectraMax M5, Molecular Devices, San Jose, CA, USA). Fluorescence for GFP expression (485 nm for excitation, 538 nm for emission, 530 nm for cut-off) and absorbance for optical cell density (600 nm) were measured. Each measurement was conducted in Corning 96-well microplates 250 µL of each culture. All sensor output was normalized to cell density (GFP fluorescence/OD_600_) at a specific time point, and for each treatment, its control group (GFP-free cells) was subtracted.

For 0–1 normalization, each value was subtracted from the minimum value of the same group of interest and divided by the difference between maximum and minimum values in the same group of interest.

### 2.4. Statistical Analysis

For statistical analysis of the dynamic range experiments, specific binding with the Hill slope method was implemented to see if the sensor responded to increasing concentrations of cadmium ions with a sigmoidal curve.

To detect and eliminate outliers, we used ROUT method with maximum desired False Discovery Rate, Q, as 1%.

One-way analysis of variance (ANOVA) or multiple t-tests were utilized to analyze columned graphs based on the group of interest using GraphPad Prism v6 software. Data were visualized with mean ± standard error mean (SEM) in each graph. At least three biological replicates were used for each analysis.

## 3. Results and Discussion

### 3.1. Construction of Recombinase-Based Cadmium Detecting Sensor

In our WCB, we first utilized a semi-specific heat shock biosensor system where constitutively expressed HspR protein negatively regulates its cognitive promoter, PdnaK containing double HspR binding motifs (IR3). HspR protein binding to the regulatory domains of the PdnaK promoter is affected by the introduction of a stress factor. However, the stress factor can be various such as heat, oxidative stress, and heavy metals [32]. To increase the specificity of such biosensors, we utilized a compound-specific biosensor as the second layer of control for heavy metal detection. Consequently, to construct a cadmium detecting sensor, we incorporated our previously engineered HSR system [26] that controls the expression of a cadmium-specific transcription factor, CadR, and a site-specific recombinase, Bxb1. Without a stress factor, the cognitive promoter of CadR, PcadA, is inactivated since its sequence is inverted in the initial circuit, yet flanked by Bxb1 recognition sites, attP, and attB (Figure 1a). In the presence of cadmium ions, HspR is released from PdnaK, allowing the expression of Bxb1 and CadR. While Bxb1 recombines the inverted PcadA promoter, CadR forms heterodimer complexes with cadmium ions and binds to PcadA [27], driving the expression of fluorescence reporter (Figure 1b).

### 3.2. Characterization of the System to Optimal Working Conditions

To begin with, we started to optimize our circuit by comparing the conditions presented in the literature for recombinase activity. One of the main objectives of this study is to characterize the incorporation of recombinases and obtain optimal working conditions of the recombinase-based cadmium sensor so that the system results in: (i) tight control of the leakiness; and (ii) shortening the response time against the requirement of a long time due to proper expression and Bxb1 recombinase activity [25]. Additionally, to systemically improve the biosensor’s dose-response behavior, we aimed to obtain low basal expression in the OFF state, whereas sensitive responsiveness is in accord with increasing cadmium ion concentrations [33,34].

**Figure 2:**
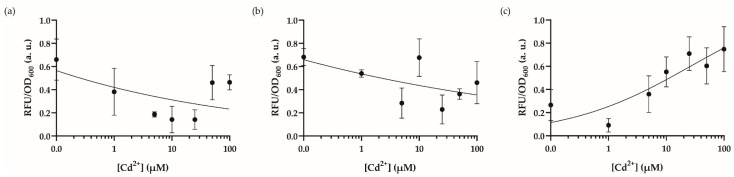
Dynamic range analysis of the engineered cadmium biosensor in different media. The dynamic range analysis of the recombinase-based cadmium sensor in (**a**) LB medium, (**b**) HMM medium, (**c**) MOPS minimal medium. Cultures were induced in 96-well plates with cadmium ion concentrations from 0–100 μM and incubated for 18 h at 30 °C in a stable incubator. The fluorescence intensity of each group was compared with each other and 0–1 normalized according to the formula stated in the Materials and Methods section. Data were visualized with mean ± standard error mean (SEM) in each graph. At least three biological replicates were used for each analysis. The point on the *y*-axis (0.0) represents the uninduced condition/background signal.

**Figure 3:**
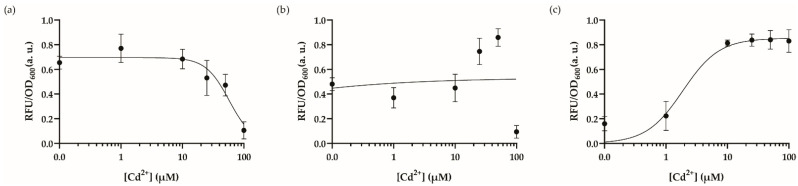
Dynamic range analysis of the engineered cadmium biosensor by changing the cell inoculum. The dynamic range analysis of the recombinase-based cadmium sensor in MOPS media with cell dilutions (**a**) 1:100, induced in 96-well plates, (**b**) 1:250, induced in 96-well plates, (**c**) 1:50, incubated at 30 °C until OD_600_ reaches to 0.4–0.6, then transferred and induced in 96-well plates. All cultures were induced with cadmium ion concentrations from 0–100 μM and incubated for 20 h at 30 °C in a stable incubator. The fluorescence intensity of each group was compared with each other and 0–1 normalized according to the formula stated in the Materials and Methods section. Data were visualized with mean ± standard error mean (SEM) in each graph. At least three biological replicates were used for each analysis. The point on the *y*-axis (0.0) represents the uninduced condition/background signal.

**Figure 4:**
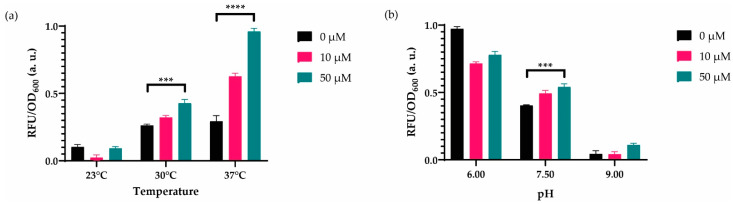
Dynamic range analyses for changing temperature and PH. (**a**) The recombinase-based biosensor was induced with 0 μM, 10 μM, and 50 μM cadmium ion in incubators, in which temperatures were 23°C, 30°C, and 37°C for 14 h. Measurements were conducted on 96-well plates. (**b**) The sensor was cultured in MOPS minimal media with pH varying 6, 7.5, and 9 for 14 h at 30°C in a stable incubator. For both (**a**) and (**b**), the initial number of cells was 1:50 of culture volume. The fluorescence intensity of each group was compared with each other and normalized according to the formula stated in the Materials and Methods section. Data were visualized with mean ± standard error mean (SEM) in each graph. At least three biological replicates were used for each analysis. For statistical analysis, multiple-t tests were implemented based on the group of interest. *** indicates *p*-value of <0.005, **** indicates *p*-value of <0.0005. Statistically nonsignificant results have no stars.

We started the system optimization with the culture media. First, we utilized Lysogeny Broth (LB) medium, yet its undefined nature resulted in poor signal output (Figure 2a), which has also been shown previously to reduce metal sensitivity in such biosensors [35]. Then, we moved on to heavy metal MOPS (HMM) minimal media (Figure 2b), however, this also provided poor growth potential for recombinase-based WCBs induced with highly toxic cadmium ions, whereas we obtained better results using MOPS minimal media; fluorescence intensity increased with respect to increasing cadmium concentrations (Figure 2c). Encompassing the MOPS minimal media for our WCB, we then tested the optimal initial number of cells before induction by changing the dilution factor for overnight cell cultures (Figure 3). We speculated that changing the initial number of cells exposed to the cadmium ion may affect the sensitivity of the cells’ stress mechanism. Hence, we compared the effects of starting cell numbers on sensor response utilizing 1:250 (Figure 3a), 1:100 (Figure 3b), and 1:50 (Figure 3c) of dilutions from overnight cultures. Consequentially, the concentration-dependent output of the cells with a different starting cell number was distinctive. Cells diluted by 1:50 and induced in the logarithmic phase (OD_600_~0.5) improved the dose-response behavior and decreased response time for later optimization steps to 14 h (Figure 4).

In addition, we initially used 30 °C as it is used as the optimal temperature for in vitro integrase reactions using linear DNA substrates [36,37]. Nevertheless, we assumed that the recombinase-based WCB might require a different temperature in our circuit design, and we incubated our sensor at 23 °C, 30 °C, and 37 °C to compare their effects. Multiple t-tests comparing 0–50 μM cadmium ion concentrations for three different temperatures have shown that recombinase-based WCB cultured in 37 °C has a greater statistical significance in the presence of the inducer (Figure 4a). Additionally, we optimized the pH of the media, keeping the initial temperature the same (Figure 4b) to optimize the sensor performance. Nevertheless, PH 7.5, corresponding to the natural MOPS minimal media pH worked the best. These overall results demonstrated that our engineered cadmium biosensor could be working better in MOPS minimal media (PH 7.5) at 37 °C.

### 3.3. Optimal Dynamic Range and Cross-Reactivity Analysis

**Figure 5:**
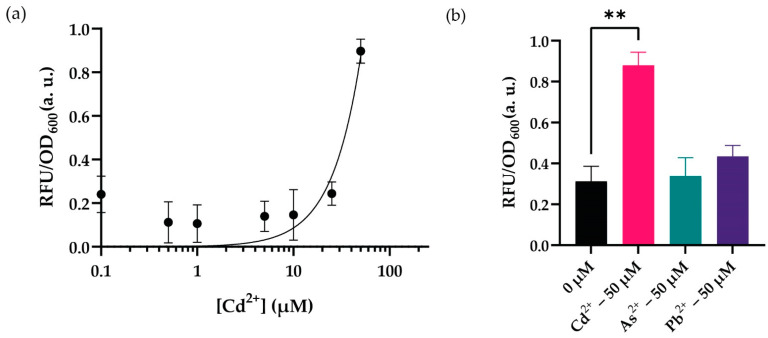
WCB activity with optimal conditions (**a**) Dynamic range analysis of the biosensor in optimal conditions with increasing concentrations (0–250 μM) of cadmium ion. The point on the *y*-axis (0.0) represents the uninduced condition/background signal. (**b**) Cross-reactivity analysis comparing biosensor’s activity with lead and arsenic ions. For statistical analysis, multiple-t tests were implemented based on the group of interest. ** indicates *p*-value of <0.005, nonsignificant results have no stars. The *p*-value for arsenic ion’s fold change between 0 μM and 50 μM is 0.99. The *p*-value for lead ion’s fold change between 0 μM and 50 μM is 0.69. For both (**a**) and (**b**), the initial number of cells was 1:50 of culture volume. The cells were cultured in MOPS minimal media with a pH of 7.5 at 37 °C for 10 h. Measurements were taken for 96-well plates. The fluorescence intensity of each group was compared with each other and normalized according to the formula stated in the Materials and Methods section. Data were visualized with mean ± standard error mean (SEM) in each graph. At least three biological replicates were used for each analysis.

Following optimization, we set up an experiment to verify our hypothesis to test our sensor in its optimal working conditions described previously. Our results showed that the sensor responded to increasing cadmium concentrations in optimal working conditions in 10 h; nevertheless, the system did not respond to cadmium concentrations lower than 50 µM (Figure 5a). The peak concentration that maximized the signal output was 50 µM cadmium. Then, we decided to test the recombinase-based sensor’s activity against other selected (II) charged heavy metal cations (i.e., arsenic and lead) since previous investigations had revealed a cross-reactive nature for the MerR family of transcription factors such as CadR [38]. The sensors seem to have a background signal for arsenic and lead ions; however, we did not observe this behavior to create a reasonable signal at 50 µM of cadmium concentration, which is the optimum response point for our system (Figure 5b). The sensor’s signal-to-noise ratio of increased cadmium ion concentration had a more significant fold change; nevertheless, testing with other heavy metal ions revealed a basal signal for our sensory system.

Although with increasing cadmium concentrations, cell growth was diminished and cells started to die as shown in the supporting information part (Figure S2), and the signal dropped with concentration change from 50 µM to 100 µM, we have detected the cadmium concentration as high as 250 µM (Figure S3). This system can be tested with other bacterial species to improve the response curve and consistency of the output and lower background, as it has been noted that detection limits can be differentiated between bacterial species [39]. Additionally, as microorganisms developed similar resistance mechanisms to a similar group of ions or molecules, there are other heavy metal ions such as Hg(II), Zn(II), and Pb(II) that have been shown to react with CadR-based biosensors [35,39]. Therefore, we need to expand our cross-reactivity analysis and incorporate parts with only specific reactions to their analytes, generated via directed evolution [40]. Finally, a feedback circuit [41] or a genetic amplifier [42] could extend our recombinase-based WCB to improve detection limits.

With our findings, we report a recombinase-based cadmium sensor utilizing a combinatory design for semi-specific and specific but cross-reactive transcriptional units. The system is optimized to get a maximum signal observed at 50 µM, yet the lower concentrations of ions could not be differentiated from the background. However, the system is promising for recombinase use in whole-cell biosensors to integrate several transcriptional units to obtain a heavy metal-specific, organism-based response. An in-depth future study to improve the characteristics in terms of selectivity and sensitivity can provide a ready-to-use biosensor for the detection of a variety of heavy metals. Here we proposed and investigated the use of a recombinase-based genetic circuit in a heavy metal biosensors system as a proof-of-concept study.

## Data Availability

Raw data is available upon request from corresponding author.

## Supplementary Materials

The following are available online at https://www.mdpi.com/article/10.3390/bios12020122/s1, Figure S1: Representative plasmid maps of the sensor, Figure S2: Cadmium-related toxicity for *Escherichia coli* DH5α strain, Figure S3: The system response with 100 µM and 250 µM, Table S1: List of oligonucleotides used in this study, Table S2: List of genetic parts and sequences used in this study.

## Abbreviations

WCBs	Whole-cell biosensors
HSR	Heat shock response
ICP-MS	Inductively coupled plasma mass spectrometry
Cd(II), Cd^2+^	Cadmium ion
As(II), As^2+^	Arsenic ion
Pb(II), Pb^2+^	Lead ion
Hg(II)	Mercury ion
Zn(II)	Zinc ion
LB	Lysogeny Broth
OD600	Optical density (600 nm)
SEM	Standard error mean
RFU	Relative Fluorescence Unit
GFP	Green Fluorescent Protein
Pc	Constitutively active promoter
MOPS	Sodium;3-(N-morpholino)propanesulfonic acid
HMM	Heavy Metal MOPS media
CadR	Cadmium-binding transcription factor of CadA
PcadA	Promoter of CadA
HspR	Heat shock protein transcriptional repressor
PdnaK	Promoter of dnaK, cognitive promoter of HspR
IR3	HspR binding motif
Bxb1	Bxb1 serine recombinase
