# Supplementary material for: A Recombinase-Based Genetic Circuit for Heavy Metal Monitoring
Source: Biosensors (Basel). 2022 Feb 16;12(2):122. doi: 10.3390/bios12020122 (PMC8870050; doi:10.3390/bios12020122)
Supplement: Supplementary file 1 [file biosensors-12-00122-s001.zip › biosensors-1524170-supplementary.pdf]

biosensors

Supplementary Information

A Recombinase-Based Genetic Circuit for Heavy Metal Monitoring

Doğuş Akboğa [1], Behide Saltepe [1], Eray Ulaş Bozkurt [1] and Urartu Özgür Şafak Şeker [1,*]

[1] UNAM- Institute of Materials Science and Nanotechnology, Bilkent University, Ankara 06800, Turkey; dogus.akboga@bilkent.edu.tr (D.A.); behide.saltepe@bilkent.edu.tr (B.S.); ulas.bozkurt@bilkent.edu.tr (E.U.B.)

* Correspondence: urartu@bilkent.edu.tr

Table S1. List of oligonucleotides used in this study.

Primer Set	Sequence (5′ - 3′)	Usage
**P$_{cadA}$-FWD**<br>**P$_{cadA}$-REV**	TGCCAAGTGAACACCCTGTAGCCAC-TATAGGGTCAAGCCGCCCG-GATGATCCTGAC<br><br>CTATAGTGGCTACAGGGTGTTCAC-TTGGCAACAGGCGTCGGGGTTT-GTACCGT	P$_{cadA}$ addition to the output module
**Bxb1-FWD**<br>**Bxb1-REV**	AGCGGGGTGCACTCATCAAAAGAG-GAGAAAGGTACCATGGGAACGGTG<br><br>TCTTCATGGTAC-CTTTCTCCTCTTTAATACGCGTTTAC-GACATCCCGGTGTG	Overhang primers for Bxb1
**CadR-FWD**<br>**CadR-REV**	GAACGGCTACACACCGGGATGTCG-TAAACGCGTATTAAAGAGGA-GAAAGGTACCA<br><br>GAGCCTTTCGTTTTATTT-GATGCCCTGCAG-TTAATGCCCGTGGCTTC	Overhang primers for CadR

Table S2. List of genetic parts and sequences used in this study.

Part	Type	Sequence (5′ - 3′)
P$_{dnaK}$	Stress Inducible Promoter	AAAAGCACAAAAAATTTTTGCATCTCCCCCTTGATGACGTGGTTTACGACCCCATTTAGTAGTCAACCGCAGTGAGTGAGTCTGCAAAAAAATGAAATTGGGCAGTTGAAACCAGACGTTTCGCCCCTATTACAGACTCACAACCACATGATGACCGAATATATAGTGGAGACGTTTAGATG
mProD	Constitutive Promoter	TCTAGATTTACAGCTAGCTCAGTCCTAGGTATAATGCTAGCTACTAGAG
P$_{cadA}$	Cadmium Inducible Promoter	TTGACTCTGTAGTTGCTACAGGGTGTGCAAT

IR3	Regulatory	GCAAGCTTGAGCGGGGTGCACTCATCA
Bxb1 attP	Regulatory	GTCGTGGTTTGTCTGGTCAACCACCGCGGTCTCAGTGGTGTACGGTACAAACCCCGAC
Bxb1 attB	Regulatory	GCCCGGATGATCCTGACGACGGAGACCGCCGTCGTCGACAAGCCGGCCGA
RBS	Regulatory	AAAGAGGAGAAA
rrnBT1	Terminator	CAAATAAAACGAAAGGCTCAGTCGAAAGACTGGGCCTTTCGTTTTATCTGTTGTTTGTCGGTGAACGCTCTCCTGAGTAGGACAAAT
GFP	CDS	ATGCGTAAAGGAGAAGAACTTTTCACTGGAGTTGTCCCAATTCTTGTTGAATTAGATGGTGATGTTAATGGGCACAAATTTTCTGTCAGTGGAGAGGGTGAAGGTGATGCAACATACGGAAAACTTACCCTTAAATTTATTTGCACTACTGGAAAACTACCTGTTCCATGGCCAACACTTGTCACTACTTTCGGTTATGGTGTTCAATGCTTTGCGAGATACCCAGATCATATGAAACAGCATGACTTTTTCAAGAGTGCCATGCCCGAAGGTTATGTACAGGAAAGAACTATATTTTTCAAAGATGACGGGAACTACAAGACACGTGCTGAAGTCAAGTTTGAAGGTGATACCCTTGTTAATAGAATCGAGTTAAAAGGTATTGATTTTAAAGAAGATGGAAACATTCTTGGACACAAATTGGAATACAACTATAACTCACACAATGTATACATCATGGCAGACAAACAAAAGAATGGAATCAAAGTTAACTTCAAAATTAGACACAACATTGAAGATGGAAGCGTTCAACTAGCAGACCATTATCAACAAAATACTCCAATTGGCGATGGCCCTGTCCTTTTACCAGACAACCATTACCTGTCCACACAATCTGCCCTTTCGAAAGATCCCAACGAAAAGAGAGACCACATGGTCCTTCTTGAGTTTGTAACAGCTGCTGGGATTACACATGGCATGGATGAACTATACAAATAA
HspR	CDS	ATGGCCAAAAATCCGAAAGATGGCGAAAGCCGCACCTTCCTGATTAGCGTGGCCGCCGAACTGGCCGGTATGCATGCCCAGACCCTGCGCACCTATGATCGTCTGGGTCTGGTGAGCCCGCGTCGTACCAGTGGTGGTGGTCGTCGTTATAGCCTGCATGATGTGGAGCTGCTGCGCCAGGTTCAGCATCTGAGCCAGGATGAAGGCGTGAATCTGGCCGGCATCAAACGCATCATTGAACTGACCAGCCAGGTGGAAGCACTGCAGAGCCGCCTGCAGGAAATGGCCGAAGAACTGGCCGTGCTGCGCGCCAATCAGCGTCGTGAAGTGGCCGTGGTGCCGAAAAGCACCGCCCTGGTGGTGTGGAAACCGCGTCGTTAA
CadR	CDS	ATGAAGATCGGAGAACTGGCCAAAGCCACCGACTGCGCCGTGGAAACCATCCGCTACTACGAGCGTGAACAGCTGCTGCCGGAGCCGGCACGCAGCGACGGCAACTACCGGCTGTACACCCAGGCCCACGTCGAGCGGCTTACCTTCATCCGCAACTGCCGCACCCTGGACATGACCCTGGATGAAATCCGCAGCCTGCTACGCCTGCGCGACAGCCCCGATGATTCGTGCGGCAGCGTCAATGCGCTGATCGACGAGCATATCGAGCATGTGCAGGCACGGATCGATGGTCTGGTGGCGTTGCAGGAACAGCTGGTGGAGCTGCGGCGGCGCTGCAATGCACAAGGGGCGGAGTGTGCGATCTTGCAGCAACTGGAGACGAACGGGGCGGTATCGGTGCCGGAAACCGAGCATTCGCATGTAGGGCGAAGCCACGGGCATTAA
Bxb1	CDS	ATGGGAACGGTGGCGCAGATGGAATTAGAAGCGATCAAAGAGCGGAACCGTTCGGCTGCGCATTTCAATATCCGCGCCGGGAAATACCGAGGATCCCTGCCGCCGTGGGGATACCTGCCTACGCGCGTGGACGGGGAGTGGCGGCTGGTGCCGGACCCTGTGCAGCGAGAGCGCATCCTCGAGGTGTATCACCGCGTCGTCGACAACCACGAGCCGCTGCATCTGGTGGCCCACGACCTGAACCGGCGTGGTGTCCTGTCGCCGAAGGACTACTTCGCGCAGCTGCAAGGCCGCGAGCCGCAGGGCCGG

GAGTGGTCGGCTACCGCGCTGAAGCGATCGATGATCTCCGAGGC
GATGCTCGGGTACGCGACTCTGAACGGTAAGACCGTCCGAGACG
ACGACGGAGCCCCGCTGGTGCGGGCTGAGCCGATCCTGACCCGT
GAGCAGCTGGAGGCGCTGCGCGCCGAGCTCGTGAAGACCTCCCG
GGCGAAGCCCGCGGTGTCTACCCCGTCGCTGCTGCTGCGGGTGTT
GTTCTGCGCGGTGTGCGGGGAGCCCGCGTACAAGTTCGCCGGGG
GAGGACGTAAGCACCCGCGCTACCGCTGCCGCTCGATGGGGTTC
CCGAAGCACTGCGGGAACGGCACGGTGGCGATGGCCGAGTGGG
ACGCGTTCTGCGAGGAGCAGGTACTGGATCTGCTCGGGGACGCG
GAGCGTCTGGAGAAAGTCTGGGTAGCGGGCTCGGACTCCGCGGT
CGAACTCGCGGAGGTGAACGCGGAGCTGGTGGACCTGACGTCGC
TGATCGGCTCCCCGGCCTACCGGGCGGGCTCTCCGCAGCGAGAA
GCACTGGATGCCCGTATTGCGGCGCTGGCCGCGCGGCAAGAGGA
GCTGGAGGGCCTGGAGGCTCGCCCGTCTGGCTGGGAGTGGCGCG
AGACCGGGCAGCGGTTCGGGGACTGGTGGCGGGAGCAGGACAC
CGCGGCAAAGAACACCTGGCTTCGGTCGATGAACGTTCGGCTGA
CGTTCGACGTCCGCGGCGGGCTGACTCGCACGATCGACTTCGGG
GATCTTCAGGAGTACGAGCAGCATCTCAGGCTCGGCAGCGTGGT
CGAACGGCTACACACCGGGATGTCGTAA

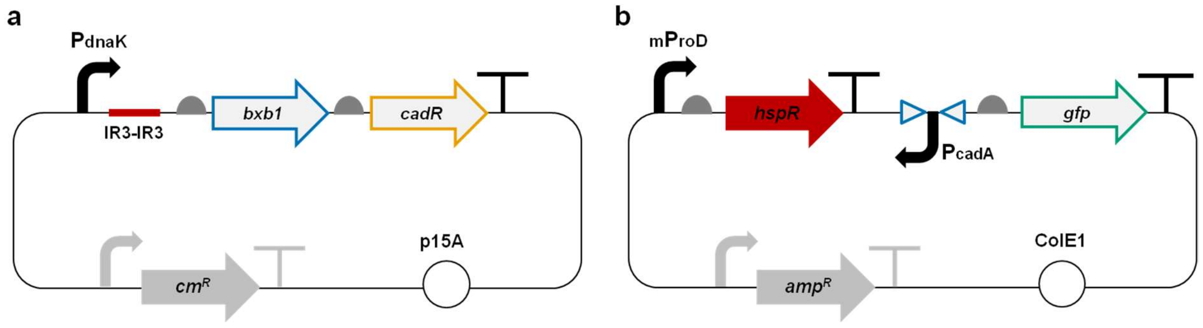

Figure S1. Representative plasmid maps of the cadmium sensor. Plasmid map containing actuating module **(a)** and sensing and output modules **(b)**. "T" represents terminator.

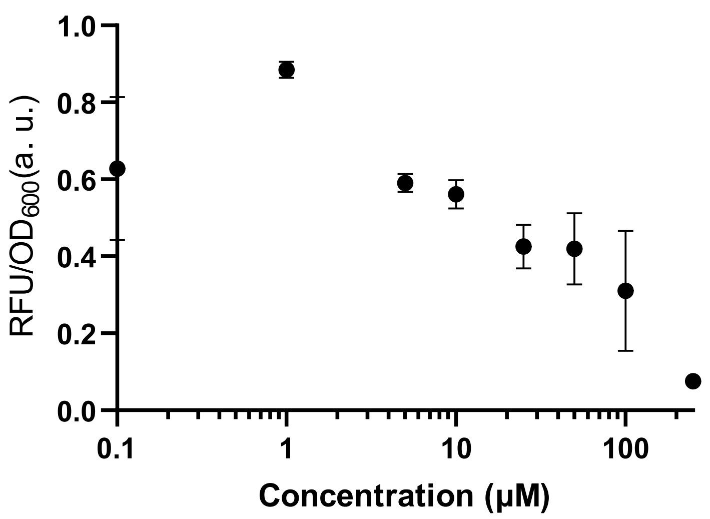

Figure S2. Cadmium-related toxicity for Escherichia coli DH5α strain. The initial number of cells was 1:50 of culture volume. The cells were cultured in MOPS minimal media with a pH of 7.5 at 37°C for 14h. Measurements were done on 96-well plates. The fluorescence intensity of each group

was compared with each other and normalized according to the formula stated in the Materials and Methods section. Data were visualized with mean ± standard error mean (SEM) in each graph. At least three biological replicates were used for each analysis.

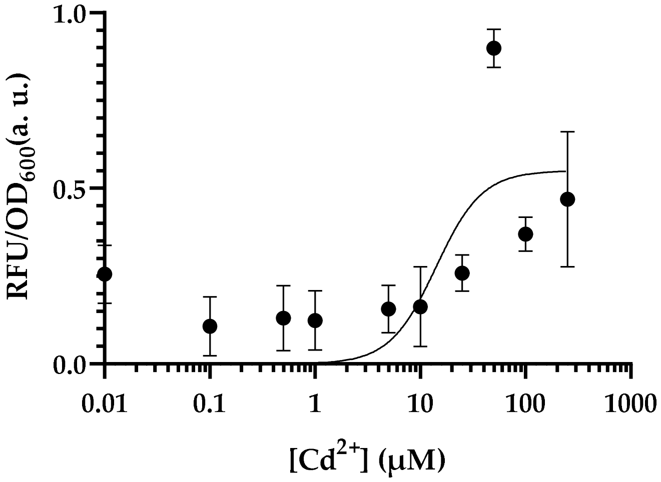

Figure S3. The system response with 100 μM and 250 μM. The initial number of cells was 1:50 of culture volume. The cells were cultured in MOPS minimal media with a pH of 7.5 at 37°C for 10h. Measurements were done on 96-well plates. The fluorescence intensity of each group was compared with each other and normalized according to the formula stated in the Materials and Methods section. Data were visualized with mean ± standard error mean (SEM) in each graph. At least three biological replicates were used for each analysis.
